# Neophobia and innovation in Critically Endangered Bali myna, *Leucopsar rothschildi*

**DOI:** 10.1098/rsos.211781

**Published:** 2022-07-20

**Authors:** Rachael Miller, Elias Garcia-Pelegrin, Emily Danby

**Affiliations:** ^1^ Department of Psychology, University of Cambridge, Cambridge, UK; ^2^ School of Life Sciences, Anglia Ruskin University, Cambridge, UK; ^3^ Department of Psychology, National University of Singapore, Singapore

**Keywords:** neophobia, problem-solving, innovation, Bali myna, conservation

## Abstract

Behavioural flexibility can impact on adaptability and survival, particularly in today's changing world, and encompasses associated components like neophobia, e.g. responses to novelty, and innovation, e.g. problem-solving. Bali myna (*Leucopsar rothschildi*) are a Critically Endangered endemic species, which are a focus of active conservation efforts, including reintroductions. Gathering behavioural data can aid in improving and developing conservation strategies, like pre-release training and individual selection for release. In 22 captive Bali myna, we tested neophobia (novel object, novel food, control conditions), innovation (bark, cup, lid conditions) and individual repeatability of latency responses in both experiments. We found effects of condition and presence of heterospecifics, including longer latencies to touch familiar food in presence than absence of novel items, and between problem-solving tasks, as well as in the presence of non-competing heterospecifics than competing heterospecifics. Age influenced neophobia, with adults showing longer latencies than juveniles. Individuals were repeatable in latency responses: (1) temporally in both experiments; (2) contextually within the innovation experiment and between experiments, as well as being consistent in approach order across experiments, suggesting stable behaviour traits. These findings are an important starting point for developing conservation behaviour related strategies in Bali myna and other similarly threatened species.

## 1. Background

Behavioural flexibility, i.e. adaptive behavioural responses to changing environments, can determine survival [1] and includes various associated components, such as innovation and neophobia. Innovation—or innovative problem-solving—can be defined as solving a novel problem or finding a different solution to a familiar problem, which influences how animals adjust to new or changing environments [2,3]. For example, invasive common myna (*Acridotheres tristis*) were more motorically innovative and tolerant of novel food than their native counterparts [4]. Neophobia, responses to novelty, is linked with life-history variation and has fitness implications [5]. Neophobia can aid in avoidance of unfamiliar dangers, though can also impact adaptation to new environments or foods, such as increased reluctance to approach novel foods [6]. How an animal responds to novelty can predict post-release outcomes during reintroductions [7]. Both neophobia and innovation may result from a combination of cognitive, including perception and learning, and non-cognitive processes, including motivation, persistence and motor diversity [8–10].

An understanding of behavioural flexibility, specifically how species and individuals respond to novelty and approach new problems [11], is vital both for behavioural research and applied conservation, particularly as the world is increasingly urbanized. Many species therefore need to adapt to human-generated environmental changes and the inevitable associated novelty [12]. Individuals that are more innovative may also be less neophobic/more neophilic (attracted to novelty), as supported by a recent meta-analysis [13]. Individuals or species with higher innovation and lower neophobia may be more adaptable in regard to coping with changing habitats, though these traits may increase chances of being trapped by humans or exposed to other dangers. Differentiating between responses to these two threats is important as populations within and between species face different levels of risk. For example, individual common myna that inhabit urban environments show lower neophobia and use novel food resources more quickly compared with those living in rural areas [14].

Furthermore, individuals may show behaviours that are temporally and contextually repeatable, or alternatively, show inconsistency in their responses [15]. This may be influenced by various factors, such as species, task or measures tested, seasonality as well as developmental and social influences [12,16]. Individual performance may also correlate across tasks. For instance, in feral pigeons (*Columba livia*) and zenaida doves (*Zenaida aurita*), latency to learn a foraging task covaried with individual neophobia level [17].

Neophobia and innovation may also be influenced by social context. For example, responses to novelty may be facilitated or inhibited by the presence of others, such as in chimpanzees (*Pan troglodytes*) [18], house sparrows (*Passer domesticus*) [19], ravens (*Corvus corax*) [20], omnivores [21], wolves (*Canis lupus*) and dogs (*Canis familiaris*) [22] and narrow-striped mongooses (*Mungotictis decemlineata*) [23]. Similarly, innovation has been found to be impacted by the presence of others, such as competitors in guppies (*Poecilia reticulata*) [24] and social transmission of a new feeding habit in canaries (*Serinus canaria*) [25].

Age may influence neophobia and innovation—with adults and juveniles differing in their responses to novelty and problem-solving capabilities within the same species. A period of higher exploration and lower neophobia is typically expected at the juvenile stage in many species [6]. For example, in human children, food neophobia is lowest in infancy and peaks between two and six years old [26]. Lower neophobia is also found in juveniles compared to adult primates (baboons, *Papio ursinus* and geladas, *Theropithecus gelada*) [27], other mammals (hyenas, *Crocuta crocuta*) [15] and birds (ravens and carrion crows, *Corvus corone*) [16], although some species show a reversed age effect (e.g. the alalā, *Corvus hawaiiensis*) [28]. Higher rates of innovation in adults than nonadults have been reported in some species across taxa, potentially relating to greater experience and manipulative competence [13].

Bali myna are a Critically Endangered species that are endemic to Bali, Indonesia. We selected this species because: (1) they are highly threatened (less than 50 adults in the wild; Birdlife.org); (2) face threats like illegal poaching for the pet trade and habitat degradation [29] that could be mitigated through behavioural research and training, which must be informed by research; (3) there is active conservation action with varying success across different sites [30], including reintroduction, which enables pre- and post-release research; (4) while there are conservation-based publications [30,31], there is currently minimal published cognitive or behavioural data on Bali myna despite a reasonably sized zoo population (approx. 950 individuals across approx. 170 institutions worldwide, with approx. 90 individuals in UK zoos; ZIMS, 2021—zims.species360.org, accessed September 2021).

As part of active conservation with Bali myna, there is a need to continually release birds to try to boost small populations, with open questions regarding ways to boost survival, such as predator/trapping avoidance and use of novel habitats and safe, new foods. A crucial first step in developing conservation behaviour approaches with Bali myna involves gathering necessary ‘baseline’ data, such as on behavioural flexibility, and demonstrating the feasibility of doing so. The next step would then be to implement these and related findings in conservation strategies, such as informing release decisions, developing training protocols with captive birds to modify cues and teach skills important for survival, like avoidance of traps and predators or attraction to safe nesting sites. As novelty responses can impact post-release outcomes in other species [7], testing novelty responses at the individual and species level can then inform pre-release training protocols. For example, targeted training to increase fear responses to traps or people where poaching is highest or to decrease neophobia by exposure to unfamiliar safe food sources in areas with low resources.

We aimed to quantify individual and species-level performance in innovation and neophobia tasks in captive Bali myna, using comparable paradigms tested in other species previously [4,6,32]. Innovation was tested through three simple problem-solving tasks: flip bark, flip cup and lift lid to obtain preferred insect reward (3 × 20 min trials per task). Neophobia was investigated through presentation of three types of novel objects and novel foods (jelly) placed beside the familiar food. The novel items were compared to the presentation of familiar food alone as the control condition (run 3 × 20 min trials per condition for individual repeatability) [15]. Furthermore, we tested whether individual performance correlated across the two experiments, i.e. whether less neophobic individuals were also quicker to approach and solve the problem-solving task(s). We tested individuals within three UK zoos, either alone, in a pair or (in one case) a group of conspecifics, and with/without heterospecifics present—some of which were competitors for food resources—as it was not possible to separate individuals for testing.

We expected that similar to other species (e.g. ravens [33]), social context would influence neophobia and innovation in Bali myna. We expected neophobia to vary between conditions and ages, with repeatability within individuals. Specifically, as in some other species, longer latencies in the novel object compared with novel food and control conditions, and in adults compared to juveniles [27,28,32]. We also expected age may influence innovation, with adults being more innovative than juveniles and, in both experiments, for individuals to be largely repeatable in their performance across rounds and conditions, as indicated in other species [13,27,28,32]. Finally, we expected that individual performance would correlate across innovation and neophobia experiments, as in other species (pigeons [34]; corvids [35]; birds and primates [36]). This study provides the first assessment of two associated components of behavioural flexibility, which may influence adaptability in Bali myna.

## 2. Methods

We pre-registered this study prior to data collection at OSF (without data analysis plan): https://osf.io/hsf43/?view_only=cac9b1cec61d44058927a65dee17d22d.

### 2.1. Subjects

Subjects were 22 captive Bali myna (10 males; 10 females; 2 unknown sex) held within three UK zoological collections (table 1). They were identifiable using coloured or metal leg rings. Subjects were 14 adults (greater than 1 year old, D.O.B. range: 2011–2019) and eight juveniles (less than 1 year old, D.O.B: mid-2020 or July 2021). Each zoo housed their birds according to their standard ethical and housing conditions, with a range of aviary sizes, though all (except one temporary inside aviary) being primarily outside, with a wide array of perching, planting and substrates available.

**Table 1.** Subject information.

UK zoo	aviary	sex (male. female. unsexed)	age (adult >1 year old; juvenile <1 year old)	group size of conspecifics	presence of heterospecifics including whether or not competitor	testing site within aviary	notes
Birdworld, Farnham	group	3.4.0	1 adult (DOB: 2018); 6 Juveniles (DOB: 2020)	7	competitor: 1 Lilac-breasted roller (*Coracias caudatus*), 3 wonga pigeon (*Leucosarcia melanoleuca*), 2 white-browed robin-chat (*Cossypha heuglini)*	main aviary	
Birdworld, Farnham	pair 1	1.1.0	adult	2	non-competitor: 2 Edward pheasant (*Lophura edwardsi*)	inside area	reared 2 chicks in July 2021 – present in aviary during round 2 of testing
Birdworld, Farnham	pair 2	1.1.0	adult	2	none	covered area of main aviary	
Birdworld, Farnham	juveniles	0.0.2	juvenile (DOB: 2021)	2	none	main aviary	tested with parents for round 2, then alone for round 3
Cotswolds Wildlife Park and Gardens	pair 1	1.1.0	adult	2	competitor: 2 white-spotted laughing thrush *(Lanthocincla bieti*), 6 azure-winged magpie (*Cyanopica cyanus*), 2 pink pigeon (*Nesoenas mayeri*), 2 Madagascar partridge (*Margaroperdix madagarensis*)	main aviary	
Cotswolds Wildlife Park and Gardens	pair 2	2.0.0	adult	2	no heterospecifics in first aviary (housing in round 1 and 2); non-competitor present in second aviary (housing in round 3): 1 pink pigeon and two Palawan peacock pheasant (*Polyplectron napoleonis*)	main aviary	moved enclosure July 2021
Waddesdon Manor	pair 1	1.1.0	adult	2	non-competitor: 1 Rothchild's peacock pheasant (*Polyplectron inopinatum*)	main aviary	
Waddesdon Manor	single 1	1.0.0	adult	1	none	main aviary	temporary single housing (new arrival)
Waddesdon Manor	single 2	0.1.0	adult	1	none	main aviary	temporary single housing (awaiting pairing with new arrival)
Waddesdon Manor	single 3	0.1.0	adult	1	none	inside house	temporary single housing (awaiting pairing or relocation)

As it was not possible to individually separate birds at any zoo due to ethical and housing constraints, as well as time restrictions, we tested the birds according to their current housing situation. There were 10 aviaries: three aviaries with single-housed birds; one aviary with a group of seven Bali myna; and the remaining six aviaries with pairs of Bali myna (male-female, except one male-male pair). Of the 10 aviaries, five also held heterospecific bird species (table 1). The heterospecifics were divided into ‘non-competitors’ and ‘competitors’, based on whether or not they routinely visited the test sites, ate Bali myna food and/or interacted with experimental apparatuses (table 1).

Participating in testing was voluntary for the birds—all available birds were present in every trial, other than the two juveniles who were only present for round 2 and 3. Data collection took place from May–July 2021, which includes the breeding season for this species (timing selected due to funding availability for this limited period). Breeding season meant that nest-boxes were present in the aviaries that housed male/female pairs for periods of testing, and one pair did successfully reproduce two chicks. It is possible that the presence of nest-boxes and attempts at reproducing may lead to increased and quicker food consumption, especially high protein foods like worms—indeed neophobia was influenced by season in rooks (*Corvus frugilegus*) [37]. Using the present dataset, we cannot test whether this impacted on neophobia in Bali myna without being able to compare to data collected entirely outside of the breeding season.

### 2.2. Pilot

Prior to testing, we visited each zoo at least twice to set up test sites, which were primarily situated where the birds were usually fed, as well as positions for video cameras (minimum of 1 m from test site, preferably further where possible, in case birds responded to the camera presence). We also recorded latencies to approach familiar food (i.e. regular diet) when fed in the morning (i.e. without any experimental manipulation) to ascertain the required length of the test trials.

### 2.3. Neophobia experiment

#### 2.3.1. Apparatus

**Figure 1.**
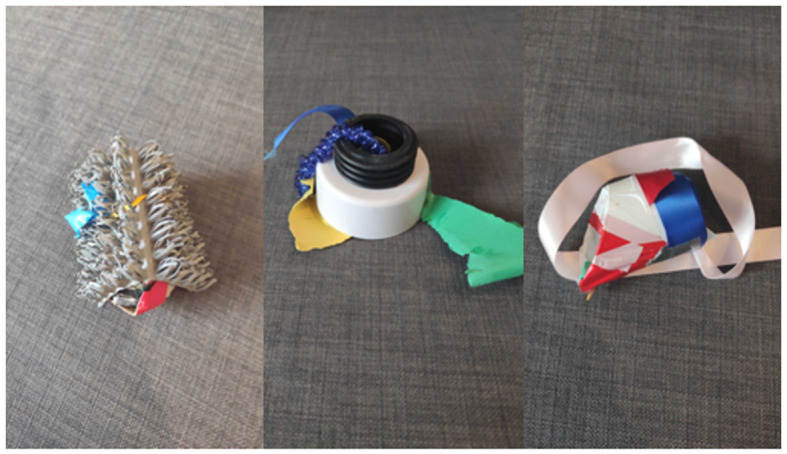
Novel objects.

We included three conditions: control (regular diet of familiar food); novel food (3 cm^3^ blocks of coloured jelly—orange, purple and green); and novel object (figure 1). The familiar food was presented in the same familiar food bowl than it would usually be served in at each aviary. Rewards were insects: mealworms (*Tenebrio molitor*), waxworms (*Galleria Mellonella*) or morio worms (*Zophobas morio*) that were added to the food bowl. The novel item was typically presented in a familiar food bowl (new bowl present in aviary for several weeks prior to testing) and always placed alongside the familiar food bowl. There were three types of novel objects—each with the same properties in terms of colours and textures—which were human-made to ensure novelty. We confirmed with keeping staff that these were suitably novel in all cases. The novel items were selected as such to be comparable with research in corvids [28,32], so the data may be useful for comparative research [38].

#### 2.3.2. Procedure

We measured behavioural responses to novel items presented alongside familiar food compared with familiar food alone. On novel item condition trials, the novel item was placed approximately 20 cm from the familiar food bowl, in the same location for each trial, therefore consistent within individual and aviary. For video coding, the trial commenced once the experimenter had left the immediate testing area (i.e. out of camera shot). Each trial lasted 20 min in total, which was determined during piloting to be sufficient time for the majority of individuals to approach the familiar food. Where there was more than one Bali myna subject in an aviary, we established more than one test site using feed sites that already existed or else following at least 2 weeks habituation and ensuring the birds fed from any new site (i.e. pair-housed aviaries received two test sites, the group-housed aviary received three test sites due to space availability). The experimenter was not present in the aviary during testing.

We ran three test ‘rounds’ in total. Within each round were three trials, one per condition (nine trials total), over 3 days, with approx. 2 weeks between rounds, therefore lasting approximately 6 weeks per zoo (table 2). Testing occurred in the morning alongside the daily presentation of their regular diet, therefore the birds were not fed prior to testing, though were not deprived and had access to any leftover food from the previous day as well as any natural foraging opportunities available like wild insects (as all included outside aviary spaces). The control trial (familiar food only) was run on day 2, with the novel food or novel object counterbalanced between day 1 or 3 across aviaries and rounds, so that the control took place within 24 h of each test condition (table 2). The main variable of interest was latency to touch familiar food, indicating the time taken for an individual to touch a familiar food when a novel item was present, with avoidance being interpreted as ‘neophobia’ (as per [6,28,32]).

**Table 2.** Order of testing. Novel object or food order counterbalanced across aviaries and rounds; control is familiar food only (i.e. no novel item present). Innovation testing occurred on the same morning as neophobia testing, after neophobia testing was complete for that day.

week	day	round number	trial number	neophobia condition	innovation condition
1	1	1	1	novel object or food 1	bark
	2		2	control	bark
	3		3	novel object or food 1	bark
4	1	2	1	novel object or food 2	cup
	2		2	control	cup
	3		3	novel object or food 2	cup
6	1	3	1	novel object or food 3	lid
	2		2	control	lid
	3		3	novel object or food 3	lid

### 2.4. Innovation experiment

#### 2.4.1. Apparatus

**Figure 2.**
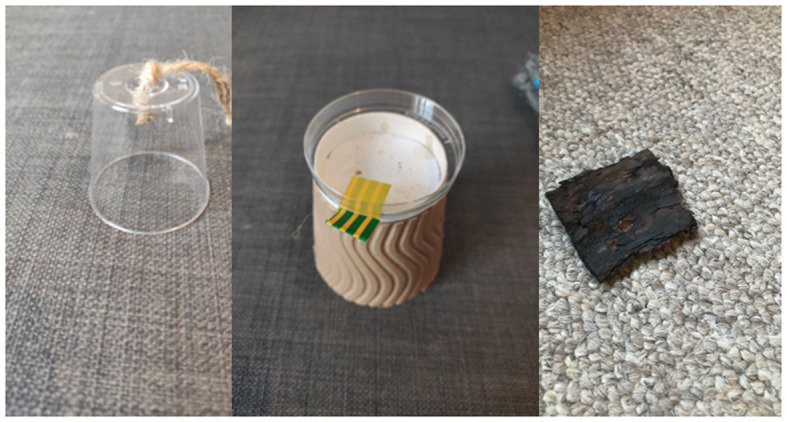
Problem-solving tasks. (1) Cup can be lifted to access worm, e.g. by pulling string or pushing cup over; (2) lid can be removed, e.g. by pushing lid or lifting tab; (3) a piece of wood bark that could be pushed or lifted to access worm.

We included three problem-solving tasks (figure 2), with a preferred insect as a reward, primarily waxworms or morio worms. Insects were humanely killed by removing their head before testing to prevent the insect from moving away.

#### 2.4.2. Procedure

Each problem-solving task was baited by the experimenter with a reward (insect) and required the subject to move an object (lid, cup, bark) to access the reward. In task 1 and 2 (lid and cup), the reward was visible, while in task 3 (bark), it was only partially visible (worm placed under bark so the tip of the body was still visible). We selected these tasks as they were relatively simple given that all subjects were unhabituated and unfamiliar with behavioural testing participation, had more than one possible method of ‘solving’ and were comparable to previous research with common myna [4]. Further, filmed reports of wild-bred juveniles learning to flip cow dung for insects, although their great, great grandparents reportedly did not do this in the aviary before release, showed they had worked it out (Donato, 2020, personal communication). The lifting/flipping behaviour is therefore likely to be part of this species behavioural repertoire.

Each task was presented 3× over 3 days, for 20 min trials per aviary, over the course of a 6-week period, with testing every 2 weeks (table 2). Innovation testing occurred in the morning after the neophobia testing for that day was complete. We presented one set of each task per subject for all aviaries. As with neophobia, the experimenter was not present in the aviary during testing, and the video was coded from when the experimenter left the test area(s). If the subject(s) solved the task within the first 5 min, the experimenter re-baited it with a new reward item. We measured: latency to approach and solve as well as frequency of peck (touching the task with bill or foot, taken as a possible indicator of interest or persistence) and solve (obtaining the baited worm).

### 2.5. Data analyses

We recorded all trials and coded all videos using Solomon Coder [39]—the primary coder (E.D.) was unfamiliar with the species and hypotheses prior to coding. We second coded 12% of videos and inter-rater reliability was strong: neophobia (Cohen's Kappa = 0.8), innovation (Cohen's Kappa = 0.82).

For the neophobia experiment, we were interested in two main questions: (1) testing effects of condition (control, novel food, novel object), round (1–3), presence of heterospecifics (none, competitor—touches Bali myna food, non-competitor—does not touch this food) and age (juvenile, adult); (2) individual repeatability over round and condition. The main dependent variable was latency to touch familiar food (0–1200 s). Analysis was run using R (v. 4.1.0) [40] and SPSS (v. 27). For Q1, we conducted a Linear Mixed Model (LMM) with a Gaussian distribution to test whether the main effects of condition, round, presence of heterospecifics and age influenced latency to touch familiar food, with aviary and individual nested in aviary as a random effect, using Tukey comparisons for *post-hoc* comparisons (package multcomp, function glht()). To test the model's assumptions, we used the DHARMa package [41]. Our model did not fail to converge, and exhibited a confidence interval of 97.5%. The assumption checks of our model evidenced no deviation from the expected distribution but showed some quantile deviations of the residuals against the predicted values. For Q2, we tested individual repeatability over time (i.e. across rounds) and over condition using intraclass correlation coefficients (ICCs) (per [28]).

For the innovation experiment, we checked whether frequency to peck (as a potential indicator of interest or persistence) correlated with frequency to solve using two-tailed Spearman's correlations on trials without including cases where both measures were zeros (73/198 trials). Although 77% of subjects interacted with the tasks at least once, the data were heavily skewed towards zero, with relatively little variance. Given the care required when using more complex analysis, such as models using small, low variance data sets, we found that mixed models were not the most suitable approach. Therefore, we used non-parametric statistics for this analysis—namely, Wilcoxon signed ranks tests and Mann-Whitney *U*-tests, with Bonferroni corrections applied for multiple comparisons. We compared condition (bark, cup, lid), presence of heterospecifics (none, competitor, non-competitor) and age (adult, juvenile) on four variables of interest: (1) latency to approach task (maximum latency being 20 min/1200 s); latency to solve; (2) frequency of peck; (3) frequency of solving. We also tested individual repeatability over time (i.e. across rounds) and over condition using ICCs using latency to approach and solve measures.

Finally, we tested whether individual performance correlated across the two experiments using intra-class correlation coefficients. As subjects were temporally repeatable in both experiments, we created mean scores across round (neophobia) or trial (innovation). We then correlated individual latency to touch familiar food in the object condition of the neophobia experiment with (1) latency to approach and (2) latency to solve in the innovation tasks using these mean scores. We used novel object (rather than novel food) in this case as it was more comparable to the novel problem-solving task context where rewards were familiar foods. We selected the latency measure for comparability across experiments, however, we note that they do not both measure responses to novelty. In the neophobia experiment, subjects were presented with each novel item only once (three novel objects; three novel foods) and over 6 weeks, whereas in the innovation experiment, subjects were repeatedly shown the same problem-solving task three times over three successive days and thus cannot be considered novel. Furthermore, we used the mean scores to check whether order of approach to the innovation tasks and neophobia tasks correlated across experiments within each aviary using ICCs.

Example video trials can be found at: https://youtu.be/roVTMDfZcwU.

## 3. Results

### 3.1. Neophobia experiment: testing effects of condition, round, presence of heterospecifics and age

**Figure 3.**
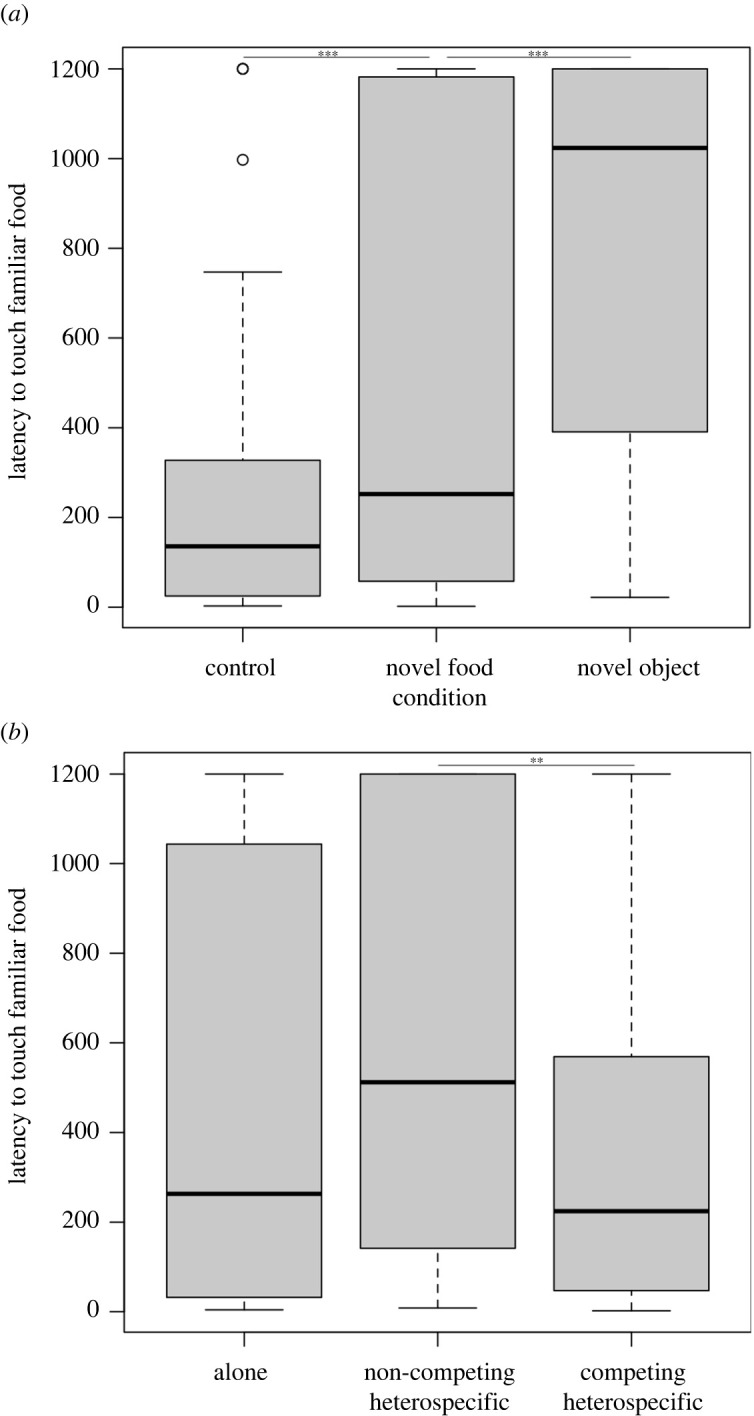
Latency to touch familiar food (seconds) differed by (*a*) condition and (*b*) presence of heterospecifics. Raw data; lines represent median. ***p < 0.001; **p < 0.01.

Latency to touch familiar food differed between conditions (LMM: *χ*^2^ = 86.533, d.f = 2, *p* < 0.001), presence of heterospecifics (*χ*^2^ = 6.901, d.f = 2, *p* = 0.032) and age (*χ*^2^ = 4.275, d.f = 1, *p* = 0.038), but not between test rounds (*χ*^2^ = 4.985, d.f = 2, *p* = 0.082). The birds took longer to touch familiar food when a novel object or novel food was present compared to the control condition (Tukey contrasts: novel object – control, *z* = 9.285, *p* < 0.001; novel food – control, *z* = 4.075, *p* < 0.001) and they took longer when a novel object was present than a novel food (*z* = 5.339, *p* < 0.001) (figure 3*a*). Across conditions, they showed longer latencies when non-competing heterospecifics were present compared with when competing heterospecifics were present (Tukey contrasts: *z* = −2.617, *p* = 0.023). There was no difference in latencies when alone compared to non-competing heterospecifics (*z* = 0.789, *p* = 0.705) or alone compared to competing heterospecifics present (*z* = −1.561, *p* = 0.258; figure 3*b*). Adults waited longer to touch familiar food than juveniles (*z* = 2.068, *p* = 0.038). Subjects touched the novel food in 3 of 62 trials (4.8%—three individuals in the ‘group’ aviary on round 3) and novel object in 0 trials, therefore latency to touch the novel items was not an informative measure for testing.

### 3.2. Neophobia experiment: individual temporal and contextual repeatability

In the neophobia experiment, we found that individuals were temporally repeatable across three test rounds (intra-class correlation coefficient: *N* = 22, ICC = 0.632, *p* < 0.001, CI = 0.435–0.768). Individuals were not contextually repeatable across novel item conditions (novel object, novel food) in their responses to novelty (ICC: *N* = 22, ICC = 0.278, *p* = 0.103, CI = −0.199–0.565). Within condition, they were temporally repeatable within the control condition, but not within the two novel item conditions (control: *N* = 22, ICC = 0.0.543, *p* < 0.02, CI = 0.038–0.805; novel object: *N* = 22, ICC = 0.287, *p* = 0.182, CI = −0.501–0.696; novel food: *N* = 22, ICC = 0.278, *p* = 0.183, CI = −0.521–0.692).

### 3.3. Innovation experiment: testing effects of condition, presence of heterospecifics and age

17 of 22 (77%) subjects approached and solved at least one trial/task. Frequency to peck correlated with frequency to solve, indicating that subjects that pecked the task more were also more likely to solve it (Spearman's correlation: trials with zeros removed: *r*_20_ = 0.302, *p* = 0.01). Latency to approach and frequency of pecking problem-solving tasks differed across conditions, as subjects waited longer to approach and pecked less frequently in the bark than cup condition (Wilcoxon signed ranks test: latency to approach – *Z* = 0.475, *p* = 0.028; frequency of peck – *Z* = −0.458, *p* = 0.036), with no difference between cup and lid (latency approach – *Z* = −0.5, *p* > 0.999; frequency peck – *Z* = 0.142, *p* > 0.999), or bark and lid tasks (latency approach – *Z* = 0.425 *p* = 0.06; frequency peck – *Z* = −0.317, *p* = 0.249). Latency to solve and frequency of solving differed across conditions, with subjects taking longer to solve and solving less frequently the lid than bark condition (Wilcoxon signed ranks test: latency to solve: *Z* = −2.527, *p* = 0.010; frequency of solving – *Z* = −2.095, *p* = 0.038), with no difference between the bark and cup (latency solve – *Z* = 01.229, *p* = 0.229; frequency solve – *Z* = −1.226, *p* = 0.262) or cup and lid (latency solve – Z = −1.224, *p* = 0.227; frequency solve – *Z* = −0.528, *p* = 0.605).

**Figure 4.**
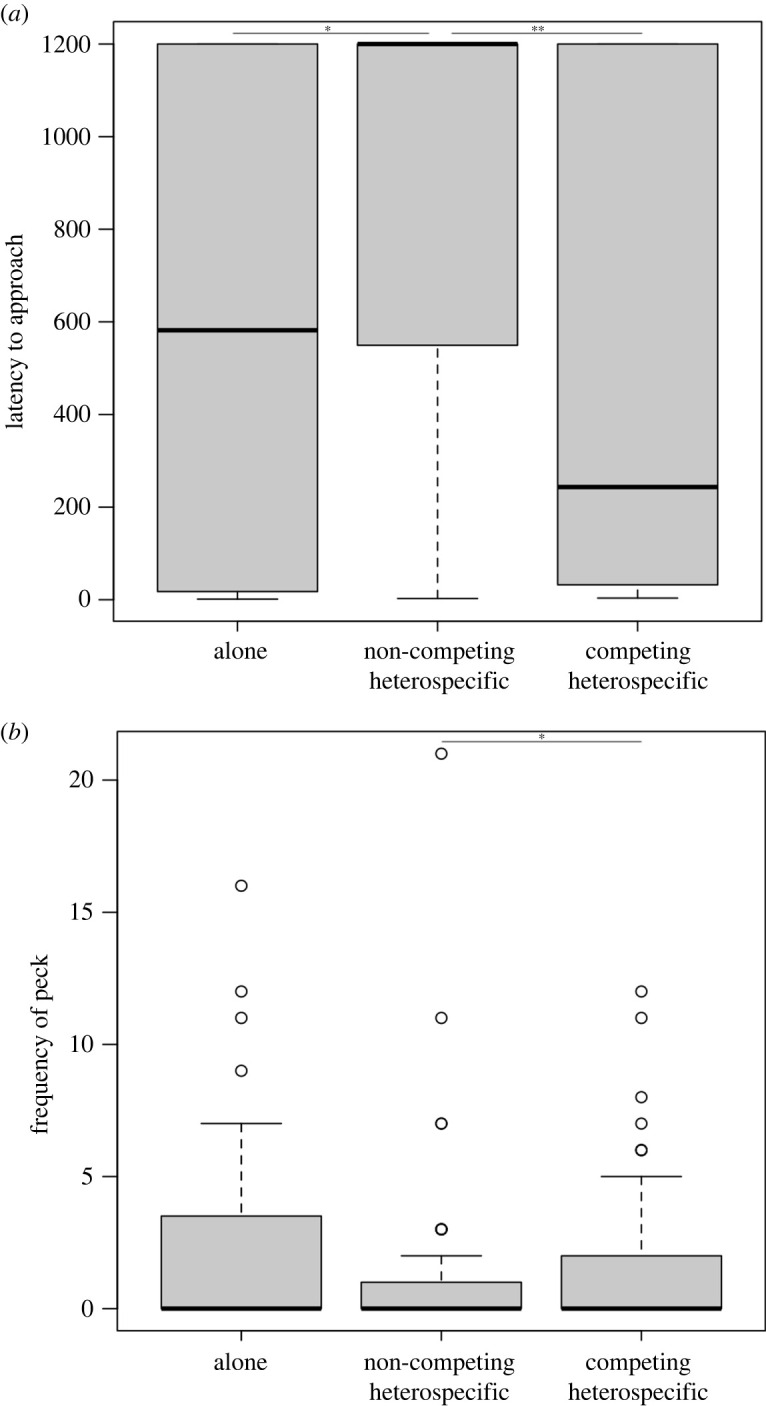
Presence of heterospecifics effect on (*a*) latency to approach (seconds) and (*b*) frequency of peck on problem-solving tasks. Raw data; lines represent median. ***p < 0.001; **p < 0.01; *p < 0.05.

Latency to approach and frequency of pecking also differed depending on whether alone, or with competing or non-competing heterospecifics present. Specifically, subjects waited longer to approach when non-competing heterospecifics were present compared with when alone (Mann-Whitney *U* test: *U* = −33.414, *p* = 0.011) or when competing heterospecifics were present (*U* = 30.315, *p* = 0.001). There was no difference between being alone compared with non-competing heterospecifics present (*U* = −3.099, *p* > 0.999; range = 0–1200 s; mean = 718.4; figure 4*a*). Subjects also pecked less when non-competing heterospecifics were present compared with competing heterospecifics (Mann-Whitney *U* test: *U* = −20.357, *p* = 0.019), with no difference compared to being alone (*U* = 20.833, *p* = 0.147) or with competing heterospecifics present (*U* = 0.475, *p* > 0.999; range 0–21 pecks; mean = 1.4; figure 4*b*). Latency to solve and frequency of solving did not differ depending on presence of heterospecifics (Kruskal-Wallis test: latency – $\chi_{2}^{2}=5.354,$ *p* = 0.069; range = 0–1200 s; mean = 936.6; frequency – $\chi_{2}^{2}=3.963,$ *p* = 0.138; range 0–4 solves; mean = 0.39). There was no difference between adults and juveniles in latency to approach (*p* = 0.806), frequency of pecking (*p* = 0.904) or frequency of solving (*p* = 0.233).

### 3.4. Innovation experiment: individual temporal and contextual repeatability

Individuals were temporally repeatable (across 1–3 trials: approach – ICC = 0.547, *p* < 0.001, CI = 0.313–0.710; solve – ICC = 0.504, *p* < 0.001, CI = 0.248–0.682) and contextually repeatable in latency to approach and solve the problem-solving tasks (across bark, cup, lid conditions: approach – ICC = 0.317, *p* = 0.040, CI = −0.048–0.570; solve – ICC = 0.598, *p* < 0.001, CI = 0.383–0.747).

### 3.5. Individual-level performance across both experiments

Using a mean score across round/trial, individual (1) latency to approach and (2) latency to solve three problem-solving tasks in the innovation experiment correlated with latency to touch familiar food in presence of novel object in the neophobia experiment (latency to approach: *n* = 20, ICC = 0.763, *p* < 0.001, CI = 0.533–0.896; latency to solve: *n* = 20, ICC = 0.748, *p* ≤ 0.001, CI = 502–889). Using the mean score, the order of approach within aviary correlated across the three problem-solving tasks and the object neophobia condition (*n* = 17, ICC = 0.915, *p* < 0.001, CI = 0.823–0.966). Note that three subjects were tested alone, and two subjects were not tested in the innovation bark task, therefore were excluded from analysis.

## 4. Discussion

We tested associative components of behavioural flexibility, specifically neophobia (latency to touch familiar food in presence of novel object or novel food) and innovation (latency to approach and solve, frequency of pecking and solving; three simple problem-solving tasks) in captive Bali myna. We found effects of condition (neophobia – control, novel object, novel food; innovation – bark, lid, cup) and presence of heterospecifics (alone, competitors or non-competitor heterospecifics) on both neophobia and innovation. Additionally, we found an effect of age (juvenile, adult) on neophobia, though not innovation. Individuals were temporally repeatable, though not contextually repeatable in their neophobia responses, while being temporally and contextually repeatable in latency responses to the innovation tasks. Individuals also showed repeatability in their latency responses and order of approaches across both experiments. These findings indicate that, for example, an individual that is quick to touch familiar food beside a novel object is also quick to approach and solve a problem-solving task, and subjects within each aviary are likely to approach the task in a similar order across trials. This study provides support for the feasibility of testing behaviour in Bali myna in future. Furthermore, while beyond the scope of the current study, it provides an important first step in gathering ‘baseline’ behavioural data that could be implemented in active conservation strategies, including pre-release training and selection of individual suitability for reintroduction.

Our findings indicating individual repeatability suggest that behavioural responses to novel objects and foods, as well as simple problem-solving foraging-based tasks, may reflect stable traits in Bali myna. Individual repeatability is crucial for any potential applications of such findings in conservation actions, particularly if using individual differences in decision-making. For example, if you selected an individual with low neophobia for release, it is important to know first whether or not this individual consistently shows low neophobia over time and context, as if not, it may not be a suitable trait for selection. Similar effects of age on neophobia have been found in other species, including birds and primates [16,27], where juveniles show lower neophobia than adults. Juvenile Bali myna may therefore be potentially more receptive to novelty exposure during pre-release training and release than adults, which is an aspect for future research. Juveniles in other species across birds, fish, mammals and reptiles have been found to derive greatest survival benefit from anti-predator training, environmental enrichment and soft release conditioning compared to unconditioned individuals [42]. Furthermore, adults in these species typically showed more variable effects of conditioning [42]. There was no difference found in innovation performance between adults and juveniles, contrary to expectations based on a recent meta-analysis, although in line with some findings, such as no age effect on propensity to innovate in chimpanzees [13,43].

Social context has been shown with other species to either facilitate or inhibit behaviours, including neophobia and exploration [16,22,44]. For instance, observing group members eating familiar food facilitates acceptance of novel foods in tufted capuchin monkeys (*Cebus apella*) [45]. In Bali myna, latency to approach and—for innovation also—frequency of pecking (i.e. interest or persistence) was influenced by the presence of others, specifically heterospecifics, in both experiments. It appears that the specific identities and/or behaviour of others present played a role, given that non-competing heterospecifics tended to inhibit Bali myna interaction behaviours, whereas the presence of competing heterospecifics (routinely interacted/ate at Bali myna food sites/stimuli) facilitated interactions. There was no influence of heterospecific presence on solving (latency nor frequency of solving) in the innovation experiment. Problem-solving performance at automated foraging devices increased with group-size in great and blue tits, particularly with the presence of an experienced bird [46]. It is possible that differing group compositions and sizes, as well as increased task complexity, may influence solving performance in Bali myna. Alternatively, solving performance may be less likely to be influenced by sociality in some species. For example, in 39 carnivore species, social complexity (i.e. solitary to large groups) did not predict problem-solving success [47].

As approach order in both experiments was consistent, i.e. that individual myna typically approached the familiar food and problem-solving tasks in a similar order, competition between conspecifics may influence behaviour less than heterospecifics. The consistent conspecific approach order may reflect a ‘socially-induced’ neophobia, where individuals wait for others to take the risk of approaching first, or alternatively related to rank, where they have to wait for access [48]. The importance of the relationship and/or identity of others, including whether they are a competitor or not, has also been shown to influence behavioural traits like exploration and neophobia, as well as innovation, in guppies, corvids, wolves and dogs [16,22,24,33]. We were unable to control or manipulate which heterospecific species were present across aviaries, however, the influence of competitors could be further explored in future. For example, the tested group of predominately juvenile Bali myna presents a rare opportunity (given that this species is most often held in pairs) for future social-based experiments, such as facilitation and tolerance around food sources with conspecifics and heterospecifics [49].

The problem-solving tasks selected were similar to one another and simple—lifting, pushing or pecking at an object to obtain a visible reward. Despite this, we found differences in responses across conditions. Specifically, longer latencies to approach and frequencies of pecking for the bark than cup condition. This is likely due to this task being the first one that was tested (i.e. test round 1). Alternatively, it may be related to the reward (insect) being less visible under the opaque bark than inside the transparent cup. Further, there were longer latencies to solve and frequencies of solving in the lid than bark condition, which may relate to task components (e.g. lift tab or push lid versus pushing or reaching under). Future work may explore understanding of object permanence, for instance, to test whether reward visibility influences behavioural responses in problem-solving tasks.

The main study limitations were uncontrollable aspects of the testing environments—including variable presence of heterospecifics, which we included as a factor in the analysis. Some heterospecifics had little recordable impact on Bali myna interactions with food or experimental stimuli (e.g. ground-dwelling species like pheasants) thus were referred to as ‘non-competitors’, while others (e.g. spotted laughing-thrush) routinely interacted with these items and thus were ‘competitors’. Interestingly, despite appearing to be quite neophobic (i.e. stronger reaction to novel items than control, particularly to novel objects), the Bali myna anecdotally frequently appeared to be one of the more dominant species in mixed-species aviaries as they displaced others (e.g. azure-winged magpies) from test/food sites. We were restricted in timing of data collection due to funding availability therefore testing overlapped with breeding season, which may impact on performance, motivation and participation. Indeed, one pair did successfully reproduce during testing, which provided a unique opportunity to test two Bali myna juveniles shortly after fledging in the presence of the parents, as well as while alone.

These were captive zoo-housed individuals limiting generalization across the species. Future work should aim to include a larger captive sample size generally as well as wild/ reintroduced birds. Behavioural flexibility, including neophobia and innovation, could be tested further using different tasks, such as novel predators, a variety of novel foods, and more complex problem-solving tasks. Similarly, as neophobia has been found to be context-specific in other species (e.g. corvids [37,50]), it would be useful to explore the flexibility and manipulations of this behavioural response to novelty. For instance, increasing (e.g. via pairing with aversive stimuli) neophobic reactions to dangerous items, like traps, or decreasing (e.g. via habituation) neophobic responses to novel safe foods prior to release. Other cognitive and behavioural aspects that are relevant to adaptability, such as social learning i.e. learning from others, would also be useful to test for applying to conservation actions. For example, social facilitation during foraging (tufted capuchin monkeys [51]; carrion crows [49]; short-tailed bats (*Carollia perspicillata*) [52]) and exploring the link between different abilities, like innovation and social learning [34,35]. Our present finding that Bali myna interactions with novelty and problem-solving tasks are influenced by social context indicates that this would be a useful avenue for future work.

## 5. Conclusion

We tested two conservation-relevant associated components of behavioural flexibility in a little-studied, Critically Endangered bird species, which could be further implemented across other species, for instance, through the ManyBirds framework [38], and used in applied sciences. Our findings help contribute to our understanding on how Bali myna and individuals react to changes in their environment. Additionally, cognitive and behavioural research contributes to conservation by encouraging positive public perception and enhanced understanding [12], which is particularly important for preventing poaching for the pet trade—a major threat to Bali myna and other species. These findings are promising starting points for the potential of future research with Bali myna and similarly threatened species, particularly those that may be available for both captive and fieldwork, with active conservation programmes, including reintroductions.

## Data Availability

The full dataset and R script are available at Figshare: doi:10.6084/m9.figshare.16974298 (private link: https://figshare.com/s/ebba9eb80bfdeb06d3dc).
